# Towards a point-of-care strip test to diagnose sickle cell anemia

**DOI:** 10.1371/journal.pone.0177732

**Published:** 2017-05-16

**Authors:** Meaghan Bond, Brady Hunt, Bailey Flynn, Petri Huhtinen, Russell Ware, Rebecca Richards-Kortum

**Affiliations:** 1 Department of Bioengineering, Rice University, Houston, TX, United States of America; 2 PerkinElmer, Turku, Finland; 3 Division of Hematology, Cincinnati Children’s Hospital Medical Center, Cincinnati, OH, United States of America; Emory University/Georgia Institute of Technology, UNITED STATES

## Abstract

A rapid test to identify patients with sickle cell disease could have important benefits in low-resource settings. Sickle cell anemia (SCA) affects about 300,000 newborns each year, the majority of whom are born in sub-Saharan Africa. Low-cost therapies are available to treat SCA, but most countries in sub-Saharan Africa lack robust neonatal screening programs needed to identify patients in need of treatment. To address this need, we developed and evaluated a competitive lateral flow assay that identifies patients with SCA (genotype HbSS) in 15 minutes using undiluted whole blood. A small volume of blood (0.5 μL– 3 μL) is mixed with antibody-coated blue latex beads in a tube and applied to the strip. Strips are then placed in a well of running buffer and allowed to run for 10 minutes. Laboratory evaluation with samples containing different proportions of hemoglobin A (HbA) and hemoglobin S (HbS) indicated that the test should enable identification of SCA patients but not persons with sickle cell trait (SCT). We evaluated the test using 41 samples from individuals with SCA, SCT, and normal blood. With visual inspection or quantitative analysis, we found a 98% accuracy when differentiating SCA from normal and SCT samples as a group (90% sensitivity and 100% specificity for identifying SCA). This work demonstrates important steps towards making a lateral flow test for hemoglobinopathies more appropriate for point-of-care use; further work is needed before the test is appropriate for clinical use.

## Introduction

A recessively inherited mutation of the beta globin gene causes sickle cell anemia (SCA); instead of normal adult hemoglobin (HbA), patients with SCA produce sickle hemoglobin (HbS). Manifestations of SCA include severe anemia, pain, and decreased immune function[1]. Homozygotes for the sickle cell gene mutation who produce only HbS (genotype SS) are affected by the disease. Heterozygotes, producing both HbA and HbS (genotype AS), have sickle cell trait (SCT) and typically show no symptoms of the disease, though they can pass on the abnormal gene to their children. In this manuscript, we use the term SCA to refer specifically to patients with genotype HbSS. Hemoglobin SC disease and sickle beta-thalassemia, involving hemoglobin S as well as other hemoglobin mutations, are other forms of sickle cell disease.

Piel et al. estimated that 312,302 babies were born with sickle cell disease in 2010 and a further 5,476,407 were born with sickle cell trait[2]. An estimated 75.5% of babies with sickle cell disease are born in sub-Saharan Africa, where there are few resources to diagnose and treat the disease[2]. Global distribution of the sickle cell mutation follows the historic distribution of malaria because persons with SCT have some protection against dying from malaria[3].

Low-resource settings lack robust newborn screening programs for SCA[4]. Only an estimated 20–50% of affected children in Africa survive to the age of five, typically succumbing to infections due to the damage caused by sickled red blood cells to the spleen[1]. Treatments such as hydroxyurea (to increase the production of fetal hemoglobin, HbF) and prophylaxis with antibiotics and immunizations (to prevent infection) are available at low cost in these areas, but clinicians do not have a simple, low-cost method to identify SCA patients in need of therapy. There is a need for a point-of-care diagnostic appropriate for newborn screening in a low-resource setting.

Isoelectric focusing (IEF) and high performance liquid chromatography (HPLC) are considered gold standards for sickle cell diagnosis, differentiating between normal, SCT, and SCA samples[5]. IEF separates proteins in a pH-gradient gel based on slight differences in isoelectric point, and HPLC separates proteins based on differing interaction times with a column. Other electrophoresis techniques or genetic analysis may also be used to diagnose SCA. While these diagnostic methods are sensitive and specific, all require expensive equipment and extensive training and are thus not appropriate for point-of-care use, especially in low resource settings.

To meet this need, several tests have been developed to screen for SCA at the point of care. Tests like SickleDex (Streck, Omaha, NE) exploit solubility differences in the HbA and HbS molecules [6]. SickleDex can discriminate SCA and SCT from normal blood, but cannot discriminate SCA from SCT. Erroneous results can be caused by sickle cell trait, severe anemia, blood from neonates with high concentrations of fetal hemoglobin (HbF), and other factors[6]. Yang et al. adapted this principle to a paper-based assay, where blood is mixed with SickleDex reagents and spotted on paper[7]. Precipitated HbS is trapped in the fibers of the paper, while free HbA wicks away from the center of the spot. The color intensity of the outer ring can be used to differentiate between individuals with SCA, SCT, and normal blood. In the United States, the method distinguished SCA from SCT and normal with 93% sensitivity and 94% specificity using visual evaluation, and distinguished SCT from normal in Cabinda, Angola with 94% sensitivity and 97% specificity (none of the samples in Cabinda had SCA)[8]. However, the dilution requirement makes the method difficult to use in low-resource settings, and the test may be difficult to expand to detecting other forms of hemoglobin (like HbC or HbF) since it relies on solubility.

Lateral flow may be appropriate as a rapid, low-cost platform to diagnose SCA. Monoclonal antibodies specific to HbA, HbS, and even HbF or HbC (another common disease-causing beta globin mutation) are available and can be used to develop immunochromatographic assays. In addition to distinguishing among SCA, SCT, and normal blood, antibody-based methods may be able to distinguish between conditions such as hemoglobin SC disease and SCT. These conditions have a similar %S content and thus cannot be distinguished by solubility methods. Recent studies examined the performance of SickleSCAN, a lateral flow assay to detect HbA, HbS, and HbC using polyclonal antibodies [9,10]. McGann et al. report a high sensitivity (98.3–99.5%) and specificity (92.5–94%) for the device to detect the presence of HbS and HbA, and 98.4% sensitivity and 98.6% specificity for distinguishing SCA from SCT and normal blood using 139 venous blood samples in the laboratory relative to a gold standard of capillary zone electrophoresis. However, SickleSCAN requires a precise measurement of blood volume, and the user must dilute the blood before adding it to the device. These requirements make the test difficult to use in low-resource settings.

Conventional sandwich immunoassays are ideal to detect antigens present at relatively low concentrations, from approximately 1 ng/mL to 500 μg/mL, though these ranges vary widely[11,12]. When antigen is present at very high concentration, it can fill all binding sites on both the capture antibody and the detection antibody, preventing a sandwich from forming and thus giving no visible line (the “hook effect” or “prozone effect”)[13]. The concentration of hemoglobin in blood ranges from approximately 0.04 g/mL to 0.20 g/mL, thus a sandwich assay to detect hemoglobin requires 40-400-fold dilution to avoid the hook effect. Alternatively, a competitive lateral flow assay can be used to detect antigens present at high concentration without dilution. In this format, antigen dried at the capture line competes with antigen in the sample to bind to a labeled detection antibody. In the absence of antigen in the sample, a signal appears at the capture line, whereas when antigen is present, signal is reduced at the capture line. Quinn et al. have recently reported a new lateral flow test (HemoTypeSC) for sickle cell using a competitive format, though their protocol still requires diluting the blood [14]. In this paper, we present a point-of-care, competitive format lateral flow test to distinguish between people with disease (SCA) and those without disease (SCT and normal) that does not require the user to dilute the sample in order to lyse the blood or visualize the results. This proof-of-concept test focuses on HbS, the most common hemoglobin mutation, with the potential for expanding to detect other hemoglobin mutations before clinical use.

## Methods

This paper describes the development and evaluation of a lateral flow strip to identify patients with sickle cell disease. The strip is first evaluated using discarded patient and volunteer blood samples to assess the strip’s performance with varying volumes of blood and with varying ratios of HbA and HbS. Then, the strip is assessed using clinical samples of SCA, SCT, and normal blood.

### Preparation of lateral flow strips

Leftover clinical blood specimens from known HbSS and HbAA donors were obtained under a protocol approved by the Institutional Review Boards at Rice University and Cincinnati Children’s Hospital. Hemoglobin was extracted from each of these samples to serve as two capture hemoglobin on the strips according to the following protocol. 1 mL PBS was added to 100 μL of whole blood to wash the red blood cells. The solution was spun for 2 min at 1,500 g and the supernatant was discarded. 400 μL of RIPA buffer (product number 89900, Thermo Scientific, Waltham, MA) with added protease and phosphatase inhibitors (product number 78440, Thermo Scientific) was mixed with the pellet to lyse the red blood cells. The tube was then spun at 13,400 g for 15 minutes, and the supernatant was collected. The protein present in the supernatant was quantified with a BCA Protein Assay (product number 23225, Thermo Scientific). Aliquots were stored at -80°C.

Before application to the lateral flow strip, the hemoglobin was thawed and diluted in PBS to 1 mg/mL. Rabbit anti-mouse IgG (SC-358919, Santa Cruz Biotechnology, Dallas, TX) was spotted at the positive control line. Proteins were applied to a nitrocellulose membrane card (HF135, Merck KGaA, Darmstadt, Germany) using a lateral flow reagent striper (Claremont Bio, Upland, CA) set at 4.5V with a flow rate of 0.15 mL/min. Strips were allowed to dry for 24 hours at room temperature. A cellulose absorbent pad (17 mm x 300 mm, Merck KGaA) and glass fiber pad (10 mm x 300 mm, Merck KGaA) were applied to the card, each overlapping the nitrocellulose by approximately 2 mm. The absorbent pad was covered with laboratory tape in order to ensure secure it to the nitrocellulose membrane. Strips were then cut to a width of 3.5 mm using a guillotine cutter (Index Cutter II, A-Point Technologies, Gibbstown, NJ). Strips were stored in tubes with desiccant for up to one month before use.

### Preparation of latex conjugate

Latex conjugation kits (400 nm, blue) were obtained from Innova Biosciences (Cambridge, UK). Anti-HbS and anti-HbA antibodies were acquired from PerkinElmer (Waltham, MA). The antibodies were first exchanged into a new buffer using the Innova Antibody Concentration and Clean Up Kit into buffer A (anti-HbS antibody) or buffer B (anti-HbA antibody). Antibodies were covalently conjugated to the latex beads according to kit directions, with anti-HbA added at a concentration of 0.4 mg/mL instead of the recommended 0.1 mg/mL. The conjugate was stored in the provided resuspension buffer with 0.1% BSA.

Strips were run according to the following basic procedure: the two populations of latex beads were mixed with a sample of blood in a tube and allowed to incubate at room temperature for approximately 5 minutes. The mixture was spotted onto the glass fiber pad of the lateral flow strip, which was placed upright in a 96-well plate containing 40 μL of PBS + 0.5% Tween-20. The strip was allowed to run for 10 minutes, and it was then removed and scanned using a flatbed scanner.

### Quantification

We developed an automated method to quantify intensity of test and control lines and to classify the sample as normal, SCT, or SCA. The strips were scanned using a flatbed scanner, and the full color images were manually cropped using ImageJ (National Institutes of Health, version 2.0.0-rc-43) [15,16]. The red channel values were extracted to reduce the signal associated with the red blood cells. The red channel values were averaged horizontally across the strip to obtain a 1-dimensional intensity profile of the strip from bottom to top. The center of the positive control line was defined to be the intensity minimum furthest along the strip. The A and S lines were located based on a fixed pixel offset from the positive control line. Background regions were defined for each line as a 30-pixel high region centered 60 pixels below the given line. Signal-to-background ratio (SBR) was calculated for each line and compared to the threshold set in the following section. The presence of signal at either of the two capture lines (HbA or HbS) determines whether the sample contained HbS, HbA, or both, and is thus consistent with either SCA, normal, or SCT blood.

### Blood samples

The study protocol was approved by the Rice University (620119, 09-67X) and Cincinnati Children’s Hospital (2013–5178) IRBs. Normal (genotype HbAA) samples were obtained from healthy volunteers under a protocol approved by the Rice University IRB (620119). Volunteers provided written informed consent prior to study participation. Blood samples (normal, SCT, and SCA) were also obtained from leftover de-identified clinical samples, for which informed consent was waived, under a protocol approved by the Rice University and Cincinnati Children’s Hospital IRBs (2013–5178). Plasma for diluting the blood was obtained from the Gulf Coast Regional Blood Bank under a protocol approved by the Rice University IRB (09-67X).

### Determining a signal-to-background ratio threshold

In order to interpret the strips quantitatively, we first determined a SBR threshold above which a line is considered present. To account for any non-specific binding of the latex beads and the increased background caused by the presence of whole blood, we determined the threshold by running strips using blood samples with a wide range of hemoglobin concentrations. 1.75 μL of anti-HbS latex conjugate, 5.25 μL of anti-HbA latex conjugate, 10.5 μL of blood were mixed in a tube. Aliquots corresponding to a volume of 3 μL of blood were added to three separate strips. Normal (genotype HbAA) and SCA (86.1% HbS, 9.9% HbF) blood was tested. Plasma was added or removed from the blood samples to create hemoglobin concentrations of approximately 16 g/dL, 12 g/dL, 8 g/dL, and 4 g/dL. The strips were scanned and analyzed to determine SBRs for each line. The average SBRs for the negative lines (A lines on the strips assessed using HbSS blood, and S lines on the strips assessed using HbAA blood) plus 3 standard deviations were used to set a SBR threshold for further experiments. Each line (A, S, and control) has a unique threshold.

### Range of blood volumes

We assessed the performance of the strips using various volumes of blood to simulate accepting unmetered blood from a patient sample. 1.75 μL of anti-HbS latex conjugate, 5.25 μL of anti-HbA latex conjugate, and various volumes of blood (0–87.5 μL) were mixed in a tube. Aliquots corresponding to a blood volume of 0.5, 1, 2, 3, 5, 10, 15, and 25 μL of blood as well as a negative control (latex conjugates only) were added to three separate strips. Blood samples used were either HbAA (16.2 g/dL) or HbSS (84.5% HbS, 9% HbF, 9.6 g/dL) for each volume tested. The strips were scanned and analyzed with the quantification algorithm.

### Ratio of HbA to HbS

We assessed strip performance for a range of ratios of HbA to HbS to determine at what ratios the strip was able to distinguish SCT from either SCA or normal blood. To have a hemoglobin concentration matching that of the HbSS blood, HbAA blood was diluted with AB+ plasma. HbSS and diluted HbAA blood of compatible blood types were mixed to simulate HbAS samples at various ratios of hemoglobin (0–100% HbA). Simulated HbAS samples rather than patient samples were used so that the ratio of the hemoglobins could be precisely controlled. 1.75 μL of anti-HbS latex conjugate, 5.25 μL of anti-HbA latex conjugate, and 10.5 μL of blood were mixed in a tube. Aliquots corresponding to a volume of 3 μL of blood were added to three separate strips. The strips were scanned and analyzed.

### Assessing accuracy with patient samples

Finally, we assessed the accuracy of the strips at diagnosing patient samples. Forty-one patient samples were acquired from leftover clinical samples of whole blood; 10 samples were SCA, 21 samples were SCT, and 10 samples were normal. High performance liquid chromatography (HPLC) was used as the gold standard diagnosis, and samples were analyzed by lateral flow within 3–9 days of collection. Hemoglobin concentration was assessed using a hematology analyzer (Ac·T diff2; Beckman Coulter, Brea, CA).

**Fig 1:**
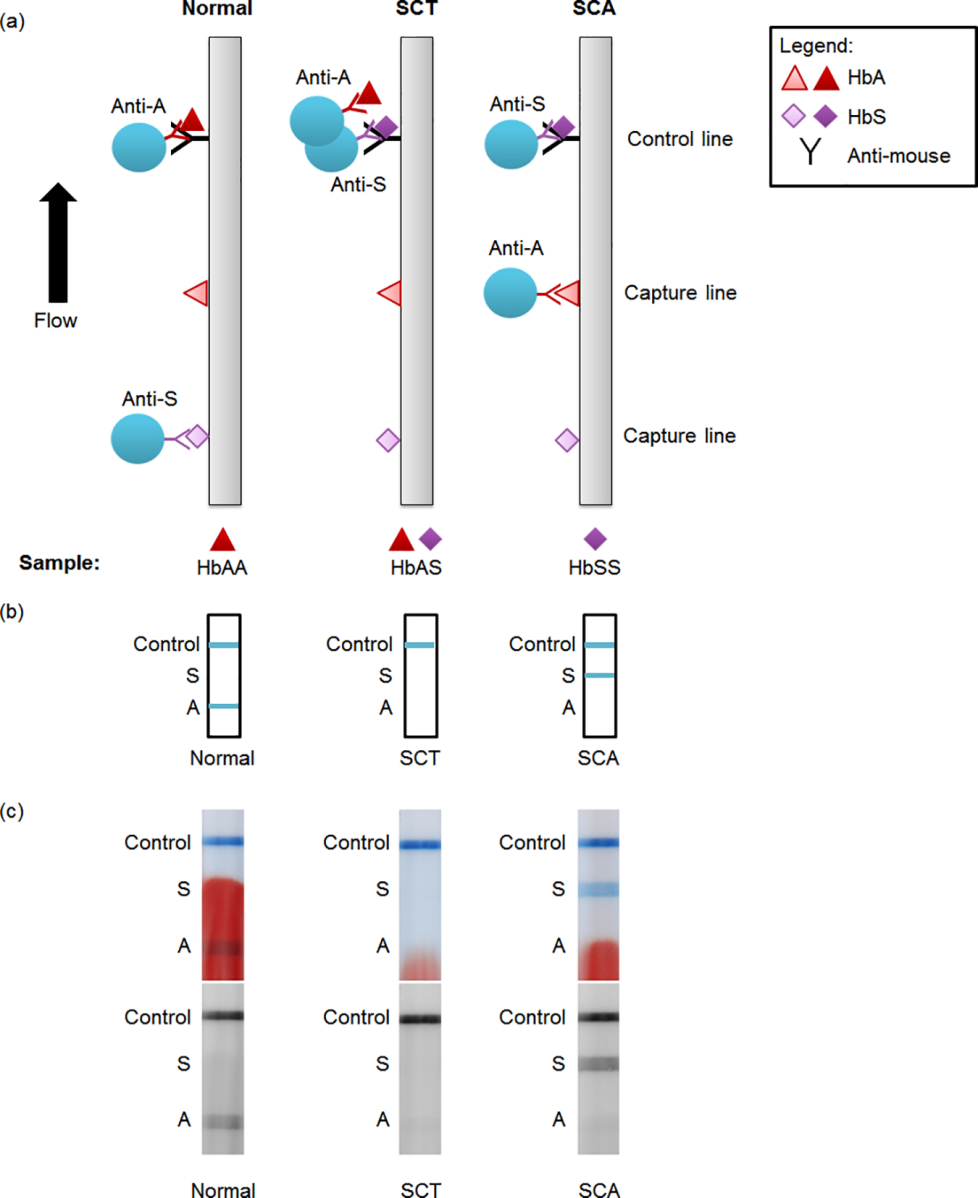
Schematic and example images of lateral flow strips. (a) Schematic of lateral flow test strip for three possible conditions: normal, sickle cell trait, and sickle cell disease blood. HbA, HbS, and an anti-mouse control antibody are dried on the strip at the capture and control lines. Two populations of latex beads (one conjugated to anti-HbA, the other to anti-HbS) and the blood sample are flowed up the strip. (b) Resulting visible readout on the strip for normal, sickle cell trait, and sickle cell disease blood. (c) Scanned images of example strips run with patient blood. Top image shows full-color scan; bottom image shows red channel of same image. Labels on the side are a guide to interpreting the competitive assay: a line present at the “A” line indicates normal, at the “S” line indicates SCA, and at neither indicates SCT. The positive control line must be present for the test to be valid.

3 μL of each whole blood sample were mixed with 0.5 μL anti-HbS latex conjugate and 1.5 μL anti-HbA latex conjugate in a tube and incubated at room temperature for 5 minutes. The strips were run and scanned. A first reader evaluated the diagnosis within 5 minutes of scanning the strip. Two additional readers evaluated the diagnosis by viewing scanned images of the strips and comparing them to images of strips with known diagnoses, such as those shown in Fig 1. Finally, the scanned images were assessed quantitatively. The lateral flow operator and all readers were blinded to the gold standard diagnosis.

## Results

Fig 1A shows a schematic of the competitive assay developed to detect SCA, and Fig 1B shows the expected readout. HbS and HbA are dried on the strip (“capture hemoglobins”), and an anti-mouse antibody serves as a positive control. In this proof-of-concept assay, sample blood is mixed in a tube with two populations of blue latex beads: one conjugated to mouse anti-HbA antibodies and the other to mouse anti-HbS antibodies. Blue latex was chosen to provide a strong contrast against a background of red hemoglobin. When the mixture is applied to the strip and the strip is placed in running buffer, the beads flow down the strip and bind to the capture proteins if no antigen is present. If sufficient antigen is present, the beads are prevented from binding to the capture protein. For example, if normal blood (containing only HbA) is applied, the sample HbA fills the binding sites of the anti-HbA latex, preventing the bead from binding to the capture HbA. The anti-HbS latex is free to bind to the capture HbS, and either type of latex may bind to the positive control line, resulting in two visible lines. Fig 1C shows example images of the strips run with normal, SCT, and SCA blood. For simplicity, throughout the paper we have called the line at which anti-HbS binds to capture HbS the “A line” because it is only visible when normal blood is present; similarly the line at which anti-HbA binds to capture HbA is called the “S line”.

### Determining a signal-to-background ratio threshold

We assessed the signal-to-background ratio of lines not expected to produce signal (the A line for strips run with SCA blood and the S line for strips run with normal blood) to establish thresholds for identifying positive lines. The strips were run with blood of various hemoglobin concentrations to obtain a wide range of background values. Setting the minimum SBR to 1.16 for the A line and to 1.06 for the S line resulted in no false positive signals. The thresholds are different because of the unique behavior of each antibody and because of the influence of the red blood, which imparts a stronger background to the A line.

### Range of blood volumes

**Fig 2:**
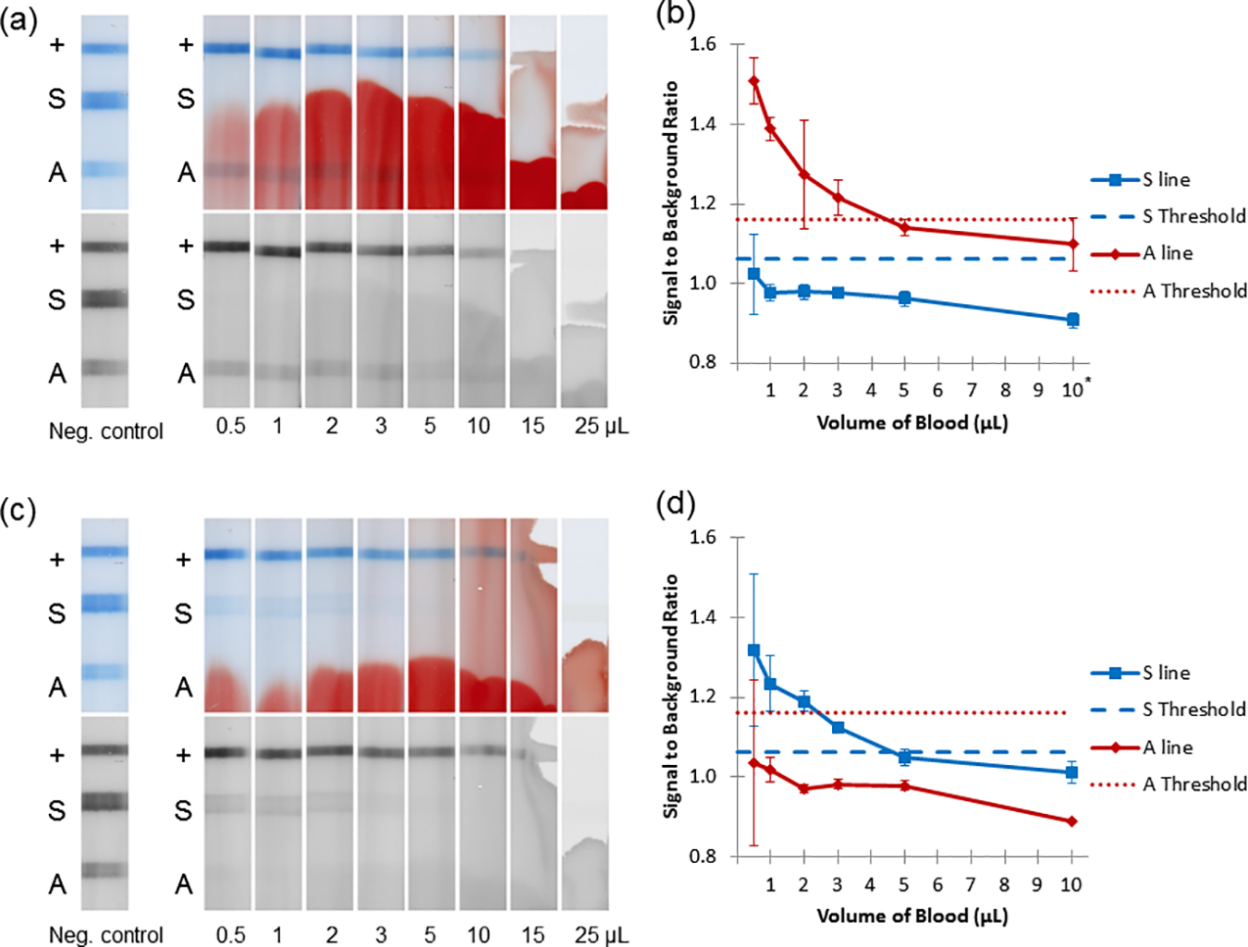
Determining the range of acceptable blood volume. (a) Representative strips run with varying amounts of normal blood, including a negative control with no blood. Top image shows full-color scan; bottom image shows red channel of the same image. (b) Signal-to-background ratio of strips in part a (normal blood) and the A threshold set by previous experiment. Error bars represent ±1 SD of three strips. *10 μL represents the average of only 2 strips. (c) Representative strips run with varying amounts of SCA blood, including a negative control with no blood. (d) Signal-to-background ratio of strips in part c (SCA blood) and the S threshold set by previous experiment.

We tested the hypothesis that a competitive assay would allow a wide range of blood volumes by assessing the performance of the strips with various volumes of normal blood (Fig 2A and 2B) and SCA blood (Fig 2C and 2D). Fig 2A shows representative images of strips run with varying volumes of normal blood. Fig 2B quantifies the signal-to-background ratio (SBR) at each test line over this range of volumes (volumes 15 μL and 25 μL were not quantitatively analyzed due to the absence of the control line, which invalidates the strip). For normal blood (Fig 2B), we expect the A and control lines to be present and the S line to be absent. The S line was below the threshold at all volumes tested. The A line is above the threshold from 0.5 μL through 3 μL, though it drops below the threshold for volumes ≥ 5 μL. This undesirable effect at high volumes of blood may be due to non-specific binding of the anti-HbS antibody to the sample hemoglobin or a dilution of the beads by the larger sample volume and a consequent decrease in signal, which is mirrored by the decrease in the intensity of the control line. These two factors (the S line and A line) combine to give an acceptable volume range of 0.5–3 μL for strips run with normal blood.

Similarly, Fig 2C shows representative images of strips with varying volumes of SCA blood, and Fig 2D quantifies the SBR at each test line. For SCA (Fig 2D), we expect the S and control lines to be present and the A line to be absent. The limit of detection is determined by the absence of the A line, which is below the threshold for all volumes tested (down to 0.5 μL). The S line is present for volumes 0.5 μL– 3 μL. The acceptable range for SCA blood is the same as that for normal blood, giving the test an acceptable range of 0.5 to 3 μL of either type of blood.

### Ratio of HbA: HbS

**Fig 3:**
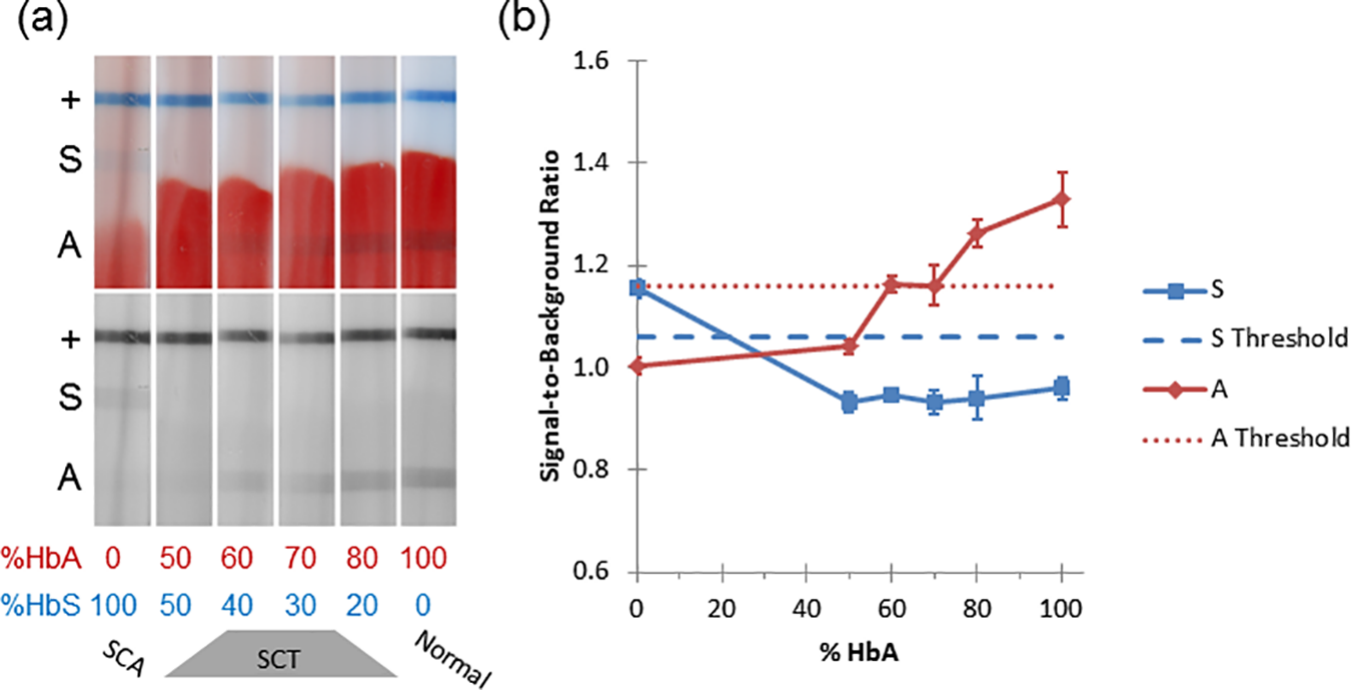
Varying ratios of HbA: HbS. (a) Representative strips run with varying ratios of HbA: HbS. Top image shows full-color scan; bottom image shows red channel of the same image. (b) Quantification of strips in part a. Dotted lines represent the thresholds. Error bars represent ±1 SD.

Fig 3 examines the effects of varying the ratio of HbA to HbS at a volume of 3 μL of blood. At 0% HbA (100% HbS), only the S line is above the threshold, and the strips are correctly classified as SCA. Clinical specimens of SCT contain 24–45% HbS [17,18]. At 50% HbS, the strips showed both lines below their respective thresholds, and the strips are classified as SCT. At 40% and 30% HbS, 3/6 strips were correctly classified as SCT and 3/6 incorrectly classified as normal. At 20% HbS the A line is above the threshold, and all the strips are incorrectly classified as normal, despite the presence of HbS. At 100% HbA, the A line is above the threshold and the strips are correctly classified as normal.

### Assessing accuracy with patient samples

The patient samples were interpreted with three methods: visual interpretation immediately after the strips were run (1 reader), visual interpretation using scans of the strips (2 readers), and automated analysis. The hemoglobin concentration of samples ranged from 4.6–16 g/dL (median of 11 g/dL).

Results from visual interpretation are shown in Table 1. (Results for each sample, including HPLC hemoglobin percentages and the hemoglobin concentration of each sample, can be found in S1 Table.) The results of the experiment assessing the ratio of HbA: HbS show that our test is not capable of reliably distinguishing normal from SCT samples at high amounts of HbA (>60% HbA). Therefore, we assessed the ability to distinguish SCA from SCT and normal samples treated as a group using patient samples. Table 1 represents the results from all 3 readers, who were concordant. We achieved an accuracy for visual interpretation of 98% (true negatives plus true positives over all tests), with 90% sensitivity and 100% specificity for identifying SCA. Sensitivity (true positives / (true positives + false negatives)) and specificity (true negatives / (true negatives + false positives)) were calculated according to standard formulae [19].

**Table 1:** Confusion matrix comparing gold standard diagnosis (HPLC) to visual interpretation results from all 3 readers, differentiating SCA samples from SCT or normal samples. The results of all three readers were concordant.

		Strip Test		
		Normal/SCT	SCA	
HPLC	Normal/SCT	31	0	**31**
	SCA	1	9	**10**
		**32**	**9**	

The results of assessing the strips quantitatively using the analysis program are shown in Table 2. One of the strips (run with SCA blood) was interpreted as invalid when both the A and S lines were above the threshold, which does not correspond to a clinical diagnosis. This strip counts as a false negative for calculating specificity, but does not appear in Table 2. Automated analysis achieves 98% accuracy with 90% sensitivity and 100% specificity for identifying SCA (Table 2).

**Table 2:** Confusion matrix comparing gold standard diagnosis (HPLC) to quantitative analysis for detection of SCA. One strip was classified as having both A and S lines present, which does not correspond to a diagnosis, and it is not included in this table.

		Strip Test		
		Normal/SCT	SCA	
HPLC	Normal/SCT	31	0	**31**
	SCA	0	9	**10**
		**31**	**9**	

## Discussion/Conclusions

We developed a competitive lateral flow test capable of identifying SCA in patient samples with 90% sensitivity and 100% specificity with either visual or automated interpretation. The test can accept 0.5–3 μL of undiluted whole blood and requires only 10 minutes to run. At small volumes, the per-strip cost of the test is approximately $2.60. The bulk of this cost is due to the Innova Biosciences latex conjugation kit; conjugating the antibodies without using this kit could bring the materials cost per strip below $0.15.

We assessed our strip with patient samples of varying hemoglobin concentration and composition. 2 of 3 readers and the quantitative analysis were able to correctly classify the sample with the lowest hemoglobin concentration (4.6 g/dL) as SCT, and all methods correctly identified the sample with the highest hemoglobin concentration (16.0 g/dL) as normal. The patient samples represented a range of HbF values (0–32.9%), and all methods correctly interpreted the SCA strip with the highest percentage of HbF (32.9%) (see specific values in S1 Table). Testing on samples with higher levels of HbF should be done before clinical use.

Our strip gives a diagnosis of SCT at 50% HbA (50% HbS) as the absence of both test lines. However, clinical specimens of SCT contain 24–45% HbS [17,18]. At these percentages, half of the strips gave a result of normal (3/6 strips), so we cannot reliably distinguish SCT from normal samples. A higher affinity anti-HbS antibody could potentially shift our ability to detect SCT into the clinical range.

Table 3 compares a number of rapid sickle cell diagnostic tools that are available or under development. While SickleDex can separate samples with SCA from normal, it cannot discriminate between samples with SCT and SCA; thus, it groups individuals who are generally asymptomatic and do not receive treatment for sickle cell disease with those experiencing disease symptoms [5]. While both the paper test described by Yang et al.[7] and SickleSCAN can distinguish among normal, SCT, and SCA blood, both tests require the user to dilute the blood prior to running the test. Our competitive lateral flow test does not reliably distinguish SCT from normal blood; however, the test can distinguish SCA from either SCT or normal blood, and can thus correctly distinguish people who typically receive treatment (SCA) from those who typically do not (SCT and normal). The competitive lateral flow strip is also the only test able to accept undiluted whole blood, though the test currently requires blood to be mixed with latex beads.

**Table 3:** Comparison of sickle cell diagnostics indicating the capability of the test to distinguish among normal, SCT, and SCA blood.

	Sample Type:	Normal	SCT	SCA	Requires Dilution?
**Diagnostic Test**	SickleDex	Normal	SCT/SCA		Yes
	Yang et al.	Normal	SCT	SCA	Yes
	SickleSCAN	Normal	SCT	SCA	Yes
	Competitive lateral flow	Normal/SCT		SCA	No
	**Condition requires treatment?**	**No**		**Yes**	

Further research needs to be done to more fully examine the effects of varying hemoglobin concentration or the percent of non-detectable hemoglobins, such as fetal hemoglobin (HbF) or other mutations like HbC. These hemoglobins dilute the detectable hemoglobins (HbA and HbS) and may also interfere through non-specific binding with the test components. However, because our test does not estimate HbS percentage to distinguish SCA from SCT but rather relies on the detection of HbA with anti-HbA antibodies, HbSC blood would theoretically return an answer of SCA (or “disease”) using our test instead of SCT or normal. Follow-up diagnostics tests such as IEF or HPLC performed after this screening test would then be able to distinguish between HbSC and HbSS blood. Further tests will be needed to verify this predicted performance for HbSC. We would expect a wide range of hemoglobin concentrations when testing healthy newborns and anemic adults affected by SCA, and similarly the percent of HbF can vary from roughly 0% to 80% when screening adults and newborns [20,21]. These additional factors may affect the amount of blood necessary to obtain a clear result using our test.

We hypothesized that a competitive assay would be able to accept a wide range of volumes. While the assay can accept undiluted blood at a range of volumes (0.5 μL– 3 μL), the range is not wide, and the upper end of this range may not be sufficiently large to accept unmeasured drops from a patient’s finger. A device like the Microsafe blood collection tube (Safe-Tec, Ivyland, PA, <$0.10 each) or an inoculating loop could assist clinicians in collecting and transferring a small amount of blood to the strip. Alternatively, simple, paper-based microfluidic approaches could be used to limit the amount of fluid applied to the device [22]. Because the test can accept a range of volumes, these methods do not have to transfer a precise volume of blood to the strip. These volume limits were only evaluated using blood at two hemoglobin concentrations, and varying hemoglobin concentrations in patient blood may affect the acceptable volume range.

Another important limitation of our test is that the latex beads and sample blood must be mixed in a tube before application to the strip. The latex should be dried into a conjugate pad before testing the device in the field. We note that this mixing step in our proof-of-concept test is of a much smaller degree (3 μL of blood with 7 μL of latex conjugate) than the dilution step required by other assays (200 fold dilution for SickleSCAN) and is for a different purpose. Other assays require dilution to (1) lyse the red blood cells, (2) reduce the amount of red color on the strip, and (3) (for sandwich assays) reduce the amount of hemoglobin on the strip to avoid false negatives due to the Hook effect. Our test does not require such dilution: (1) the test does not intentionally lyse the blood, yet sufficient hemoglobin interacts with the antibodies, (2) the blue color of the latex beads provides strong contrast even in the presence of significant red color from blood, and (3) our choice of a competitive assay allows the strip to accept a significantly higher concentration of hemoglobin than a sandwich assay. In its final form, the test will have latex beads dried into a conjugate pad and require no additional mixing step from the user.

Like other point-of-care tests for sickle cell anemia, our test will not be able to diagnose the condition in the presence of large quantities of transfused normal blood. The accuracy of the strip should be assessed in areas of low and high prevalence for SCA, as well as under conditions of varying heat and humidity. Special attention should be paid to the stability of the antibodies and capture proteins in conditions of high heat and humidity, where SCA tests are most needed, as these conditions can degrade proteins. Given sensitive and specific antibodies, the design of this strip could be expanded to detect other hemoglobin variants such as HbF and HbC. The approach described here (a competitive assay and high-contrast blue beads) combined with the higher affinity antibodies of either Quinn et al. or Sickle Scan may result in a lateral flow test that could accept a much wider range of blood volumes without dilution, thus eliminating several steps for using the assay. These changes could potentially reduce cost, improve ease-of-use, and expand access of this needed test.

This competitive assay shows promise for identifying SCA (90% sensitivity, 100% specificity), but needs improvement to identify SCT samples and to be used in the field.

## Supporting information

S1 Table Patient sample interpretations by visual readers (Readers 1–3), quantitative analysis, and the gold standard diagnosis determined by HPLC with percents of HbA, HbF, and HbS. Hemoglobin concentration was measured with a hematology analyzer.
(DOCX)
