# Supplementary material for: Towards a point-of-care strip test to diagnose sickle cell anemia
Source: PLoS One. 2017 May 16;12(5):e0177732. doi: 10.1371/journal.pone.0177732 (PMC5433739; doi:10.1371/journal.pone.0177732)
Supplement: S1 Table — Hemoglobin concentration was measured with a hematology analyzer. (DOCX) [file pone.0177732.s001.docx]

**Supplemental**

**S1 Table.** Patient sample interpretations by visual readers (Readers 1-3), quantitative analysis, and the gold standard diagnosis determined by HPLC with percents of HbA, HbF, and HbS. Hemoglobin concentration was measured with a hematology analyzer.

| Strip # | Reader 1 | Reader 2 | Reader 3 | Quantitative Analysis | HPLC | %HbA by HPLC | %HbF by HPLC | %HbS by HPLC | [Hb] (g/dL) |
| --- | --- | --- | --- | --- | --- | --- | --- | --- | --- |
| 1 | SS | SS | SS | SS | SS | 0 | 13.8 | 83.3 | 8.3 |
| 2 | AA | AA | AA | AA | AS | 91.9 | 1.2 | 4.2 | 10.8 |
| 3 | SS | SS | SS | SS | SS | 82.7 | 13.9 | 82.7 | 6.9 |
| 4 | AS | AS | AS | AS | AS | 16.5 | 4.9 | 75.6 | 5.2 |
| 5 | SS | SS | SS | SS | SS | 0 | 29 | 67.6 | 8.1 |
| 6 | AA | AS | AS | AS | AS | 75.3 | 1.3 | 20.6 | 10.1 |
| 7 | AA | AS | AS | AS | AS | 74 | 1.2 | 22.2 | 7.7 |
| 8 | AA | AS | AS | AS | AS | 11.7 | 8.5 | 72.4 | 4.6 |
| 9 | SS | SS | SS | - | SS | 0 | 29.1 | 61.6 | 8 |
| 10 | AA | AA | AA | AA | AS | 37.6 | 17.2 | 40.1 | 9.7 |
| 11 | AA | AA | AA | AA | AS | 79.2 | 7.8 | 2 | 9 |
| 12 | AA | AA | AA | AA | AS | 67.8 | 0 | 15.4 | 9.2 |
| 13 | SS | SS | SS | SS | SS | 0 | 31.8 | 60.2 | 7 |
| 14 | AA | AA | AA | AA | AS | 49.7 | 0 | 39.3 | 13.9 |
| 15 | AA | AA | AA | AA | AS | 55.9 | 2 | 28.4 | 11.5 |
| 16 | AA | AA | AA | AA | AS | 67.6 | 2.4 | 27.1 | 8.6 |
| 17 | AA | AA | AA | AA | AA | ~97 |  | 0 | 15.5 |
| 18 | AA | AA | AA | AA | AA | ~97 |  | 0 | 11.9 |
| 19 | AA | AA | AA | AA | AA | ~97 |  | 0 | 12.5 |
| 20 | AA | AA | AA | AA | AA | ~97 |  | 0 | 15.4 |
| 21 | AA | AA | AA | AA | AA | ~97 |  | 0 | 12.7 |
| 22 | AA | AA | AA | AA | AA | ~97 |  | 0 | 11.4 |
| 23 | AA | AA | AA | AA | AA | ~97 |  | 0 | 14.7 |
| 24 | AA | AA | AA | AA | AA | ~97 |  | 0 | 16 |
| 25 | AA | AA | AA | AA | AA | ~97 |  | 0 | 11.1 |
| 26 | AA | AA | AA | AA | AA | ~97 |  | 0 | 11.4 |
| 27 | SS | SS | SS | SS | SS | 0 | 20.5 | 77.2 | 6 |
| 28 | AS | AS | AS | SS | SS | 0 | 21.2 | 75.8 | 7.5 |
| 29 | SS | SS | SS | SS | SS | 0 | 11.3 | 84.8 | 8.4 |
| 30 | SS | SS | SS | SS | SS | 0 | 14.3 | 82.6 | 8.5 |
| 31 | SS | SS | SS | SS | SS | 0 | 32.9 | 64.8 | 7.6 |
| 32 | AA | AA | AA | AA | AS |  |  | 13.6 | 11 |
| 33 | AA | AS | AS | AS | AS |  |  | 37.8 | 8.5 |
| 34 | AA | AS | AS | AS | AS |  |  | 10.3 | 12.6 |
| 35 | AA | AS | AS | AS | AS |  |  | 11.1 | 11.5 |
| 36 | AA | AA | AA | AA | AS |  |  | 36.2 | 12.1 |
| 37 | AA | AA | AA | AA | AS |  |  | 20.1 | 11 |
| 38 | AA | AA | AA | AA | AS |  |  | 1 | 12.1 |
| 39 | AA | AA | AA | AA | AS |  |  | 7.7 | 11.8 |
| 40 | AA | AA | AA | AA | AS |  |  | 19.7 | 16 |
| 41 | AA | AA | AA | AA | AS |  |  | 14.2 | 13.2 |
